# A convolutional deep learning model for improving mammographic breast-microcalcification diagnosis

**DOI:** 10.1038/s41598-021-03516-0

**Published:** 2021-12-14

**Authors:** Daesung Kang, Hye Mi Gweon, Na Lae Eun, Ji Hyun Youk, Jeong-Ah Kim, Eun Ju Son

**Affiliations:** 1 Department of Healthcare Information Technology, Inje University, Gimhae, Republic of Korea; 2 Department of Radiology, Gangnam Severance Hospital, Yonsei University College of Medicine, 211 Eonju-ro, Gangnam-gu, Seoul, 06273 Republic of Korea

**Keywords:** Medical research, Oncology

## Abstract

This study aimed to assess the diagnostic performance of deep convolutional neural networks (DCNNs) in classifying breast microcalcification in screening mammograms. To this end, 1579 mammographic images were collected retrospectively from patients exhibiting suspicious microcalcification in screening mammograms between July 2007 and December 2019. Five pre-trained DCNN models and an ensemble model were used to classify the microcalcifications as either malignant or benign. Approximately one million images from the ImageNet database had been used to train the five DCNN models. Herein, 1121 mammographic images were used for individual model fine-tuning, 198 for validation, and 260 for testing. Gradient-weighted class activation mapping (Grad-CAM) was used to confirm the validity of the DCNN models in highlighting the microcalcification regions most critical for determining the final class. The ensemble model yielded the best AUC (0.856). The DenseNet-201 model achieved the best sensitivity (82.47%) and negative predictive value (NPV; 86.92%). The ResNet-101 model yielded the best accuracy (81.54%), specificity (91.41%), and positive predictive value (PPV; 81.82%). The high PPV and specificity achieved by the ResNet-101 model, in particular, demonstrated the model effectiveness in microcalcification diagnosis, which, in turn, may considerably help reduce unnecessary biopsies.

## Introduction

Mammography is an established method for the early diagnosis of breast cancer. Microcalcifications are vital markers for early breast cancer diagnosis via mammography; they facilitate nonpalpable breast cancer diagnosis of up to 55% of lesions^1,2^. The breast imaging-reporting and data system (BI-RADS) for mammography interpretation has been used extensively to standardize the classification of mammographic findings^3^. However, substantial inter-reader variability exists, and the positive predictive value (PPV) for mammographic microcalcification usually does not exceed 30%^3–6^. Therefore, a biopsy is frequently performed after a mammography, even though most microcalcifications tend to be benign. These unnecessary biopsies cost time and money, and may cause discomfort for some patients. Thus, the improvement of radiographic-diagnosis accuracy to increase the PPV of screening mammography is essential.

Deep learning methods have facilitated dramatic advances in the fields of computer vision and image analysis. The availability of large datasets and sizable computing power has boosted improvements in the diagnostic performance of deep convolutional neural networks (DCNNs) in several medical fields^7,8^. In particular, recent studies have reported that applying DCNNs for the detection and classification of microcalcifications and masses in mammograms increases classification accuracy^9–12^. Considering the small size and unobtrusive characteristics of microcalcifications in mammograms, we hypothesize that DCNNs can provide additional indicators for appropriate classification and diagnostic accuracies exceeding those achieved by expert radiologists. However, training a deep learning model requires a large amount of labeled data, which is scarce in clinical practice. Moreover, training a DCNN from scratch requires heavy computational power, large memory resources, and considerable time; furthermore, the use of limited data may lead to overfitting.

The transfer learning method helps overcome these challenges—a model pre-trained on the ImageNet dataset can be fine-tuned for application in other tasks^13^. Furthermore, the DCNN model performance can be validated using the gradient-weighted class activation mapping (Grad-CAM) method, which calculates the weighted sum of the feature map in each convolutional layer^14^.

Therefore, in this study, we fine-tuned the weights of five pre-trained deep learning models instead of training DCNN models from scratch and used these models to assess the diagnostic performance of DCNNs for classifying suspicious breast microcalcifications in screening mammograms. Grad-CAM was used to confirm the validity of the DCNN models.

## Results

**Figure 1:**
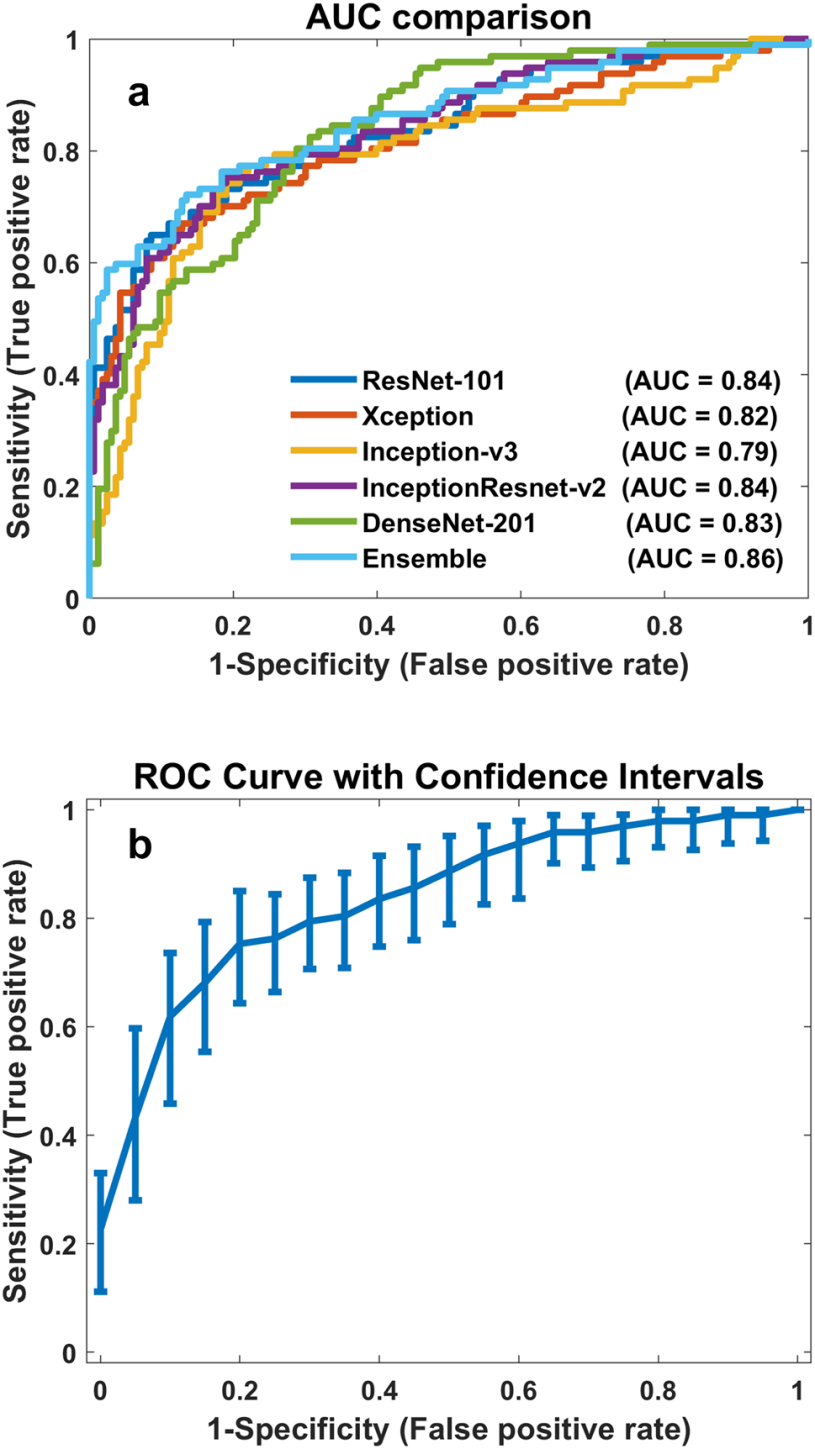
Performance of DCNN models. (**a**) Comparison of the AUCs of the five DCNN models. (**b**) ROC curve with a confidence interval (CI) for Inception-ResNet-v2, computed by generating 1000 bootstrap replicas.

Of all 1579 microcalcifications observed, 589 (37.3%) were histologically proven as malignant lesions. Table 1 lists the diagnostic performance measures of the five DCNN models at a learning rate of 1e−4. The best area under the receiver operating characteristic (ROC) curve (AUC) among all the models (0.856) was achieved by the ensemble model. The best sensitivity (82.47%) and NPV (86.92%) were obtained by the DenseNet-201 model, and the best accuracy (81.54%), specificity (91.41%), and PPV (81.82%) were achieved by the ResNet-101 model. The AUC of the DCNN models was statistically significantly different from that of the ensemble model, based on the DeLong test. The sensitivity, specificity, and PPV obtained via the generalized estimating equation (GEE) method exhibited a statistically significant difference from those obtained via the DCNN and ensemble models. The diagnostic performances (accuracy, AUC, sensitivity, specificity, PPV, NPV) of the five DCNN models are depicted in Fig. 1. The results obtained at a 1e−5 learning rate are described in the Supplementary materials. The *p* values listed in Table 1 are overall *p* values that show the overall difference between each DCNN model and the ensemble model. As the overall *p* values indicated a significant difference, pairwise comparisons were performed to calculate the *p* value for each pair of models. The pairwise diagnostic performance comparisons of the DCNN models at the 1e−4 and 1e−5 learning rates are presented in the Supplementary materials.

**Table 1:** Diagnostic performance of DCNN models (learning rate = 1e−4).

DCNN models	Cut-off point	AUC (95% CI)	Sensitivity (95% CI)	Specificity (95% CI)	Accuracy (95% CI)	PPV (95% CI)	NPV (95% CI)
ResNet-101	> 0.056	0.837 (0.784–0.890)	64.95 (55.45–74.45)	91.41 (87.11–95.71)	81.54 (76.82–86.26)	81.82 (73.21–90.43)	81.42 (75.78–87.06)
Xception	> 0.204	0.817 (0.760–0.874)	65.98 (56.55–75.41)	88.34 (83.41–93.27)	80.00 (75.14–84.86)	77.11 (68.07–86.15)	81.36 (75.62–87.10)
Inception-v3	> 0.168	0.792 (0.731–0.853)	77.32 (68.99–85.65)	77.91 (71.54–84.28)	77.69 (72.63–82.75)	67.57 (58.86–76.28)	85.23 (79.53–90.93)
Inception- ResNet-v2	> 0.276	0.838 (0.787–0.889)	75.26 (66.67–83.85)	80.98 (74.96–87.00)	78.85 (73.89–83.81)	70.19 (61.40–78.98)	84.62 (78.96–90.28)
DenseNet-201	> 0.017	0.832 (0.782–0.881)	82.47 (74.90–90.04)	69.33 (62.25–76.41)	74.23 (68.91–79.55)	61.54 (53.18–69.90)	86.92 (81.12–92.72)
Ensemble	> 0.247	0.856 (0.806–0.907)	72.16 (63.24–81.08)	86.50 (81.25–91.75)	81.15 (76.40–85.90)	76.09 (67.37–84.81)	83.93 (78.38–89.48)
*p* values		< .0001	0.0011	< .0001	0.0870	< .0001	0.1293

**Figure 2:**
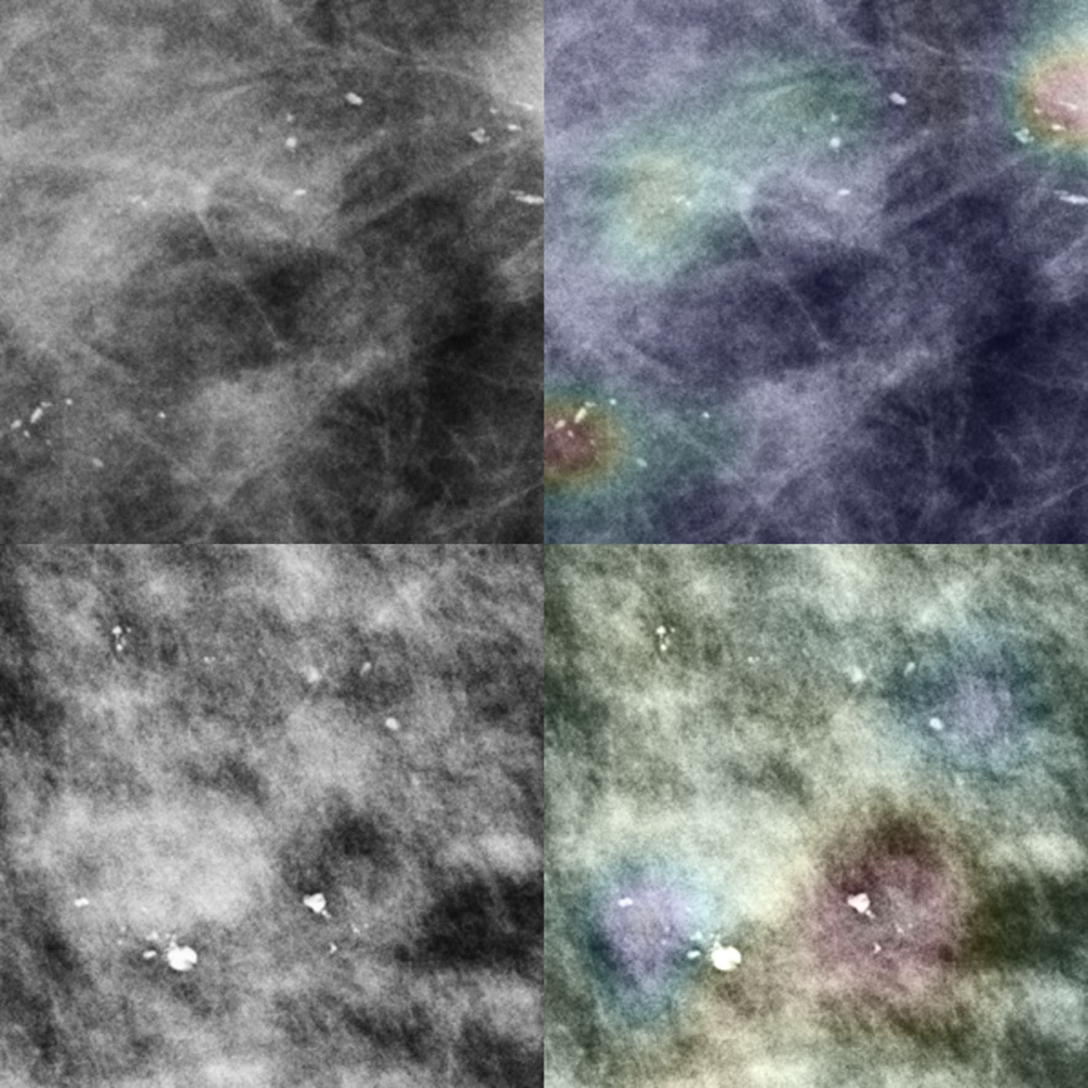
Left column depicts the original malignant microcalcification images. The right column depicts the heatmaps generated via Grad-CAM by the Inception-ResNet-v2 model overlaid on the original images. The upper- and lower-left images illustrate segmental coarse heterogeneous microcalcification. The upper and lower right images are the true positive (TP) and false negative (FN) images, respectively. The red and blue areas show the activated and less-activated regions, respectively.

**Figure 3:**
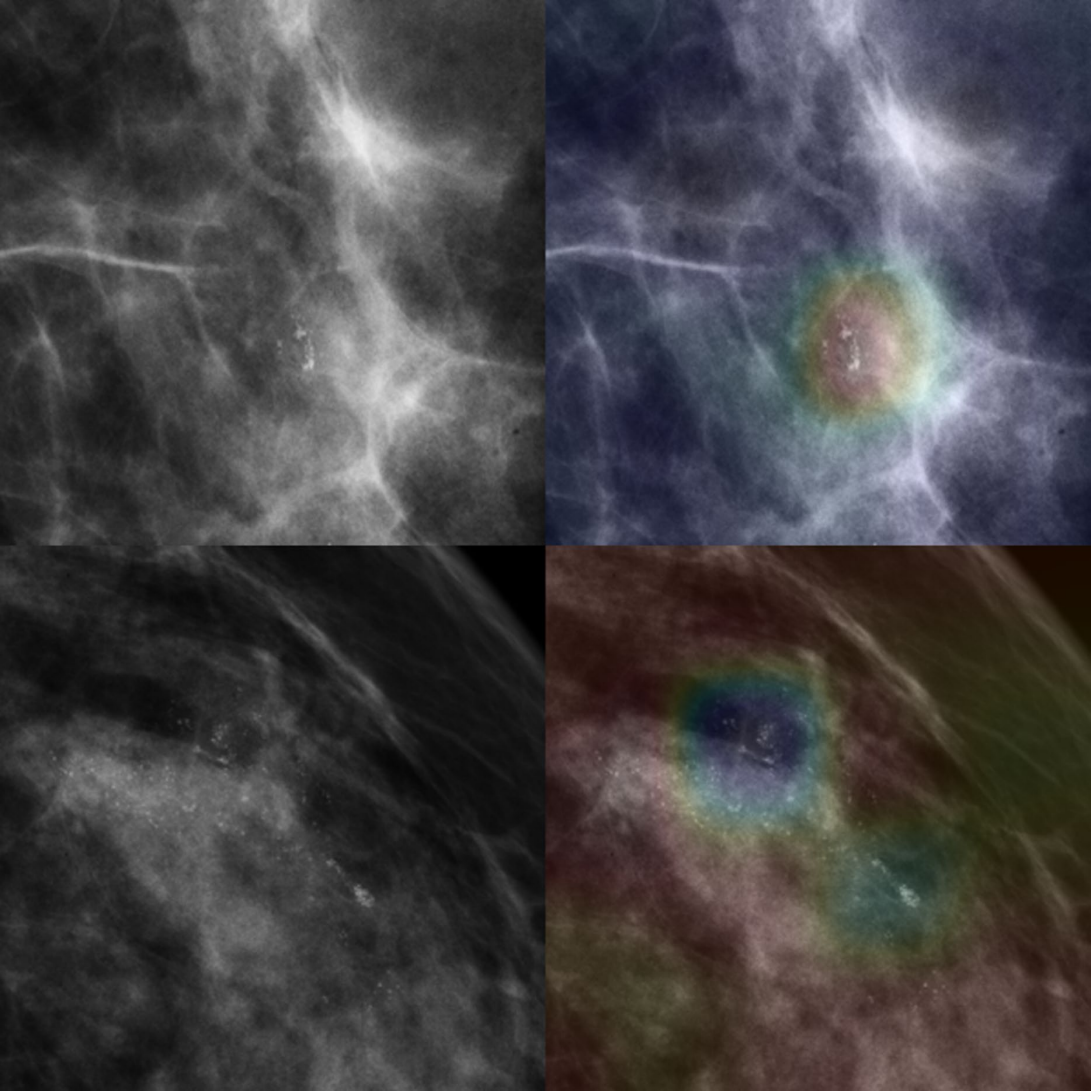
Left column comprises the original benign microcalcification images. The right column shows the Grad-CAM results overlaid on the heatmap on the original images. The upper-left image shows grouped amorphous microcalcification and lower left image shows segmental amorphous microcalcification. The upper-right image illustrates a true negative (TN); the lower-right, a false positive (FP). The Grad-CAM results were generated by the Inception-ResNet-v2 model. The red and blue areas depict the activated and less-activated regions, respectively.

The Grad-CAM method generated heatmaps, which were activated by the model to provide evidence concerning the malignancy or benignity of microcalcification regions. Figures 2 and 3 depict the Grad-CAM heatmaps generated by the Inception-ResNet-v2 model. The Grad-CAM depicts highlighted areas that were positive in predicting malignant or benign microcalcification, with areas of strong emphasis marked in red and areas of weak emphasis in blue. The upper-right heatmaps in Figs. 2 and 3 show that the red areas are important for determining the malignancy and benignity of microcalcification. In the cases wherein the DCNN model misclassified images, we could recognize that the DCNN model focused on unsuitable areas, such as those shown in the lower-right images of Figs. 2 and 3.

## Discussion

In this study, we investigated the diagnostic performance of DCNNs for patients with suspicious microcalcification in screening mammograms. The ensemble model AUC was 0.856. The specificity and PPV of ResNet-101 were 91.41% and 81.82%, respectively. The results of this study suggest that DCNNs can help radiologists determine the malignancy or benignity of microcalcifications. Earlier studies reported the existence of substantial inter-reader variability, and the PPV for mammographic microcalcifications in these studies usually did not exceed 30%^3–6^; however, the ResNet-101 PPV obtained herein (81.82%) was considerably higher. This, along with the high specificity (91.41%) of ResNet-101, can be essential for reducing the need for the unnecessary biopsies burdening patients.

Transfer learning is commonly used in deep learning applications. It has been very effective in the medical domain, wherein the amount of data is generally limited^13,15^. In this study, we utilized five state-of-the-art DCNN models: ResNet-101, Xception, Inception-v3, Inception-ResNet-v2, and DenseNet-201^16–20^. Each model was pre-trained on ImageNet^21^, and the resultant model weights were fine-tuned, instead of training new models from scratch. Through transfer learning, the microcalcification classification performance of the fine-tuned DCNNs could be assessed to improve the differential diagnosis of breast cancer and reduce false positive diagnoses.

The interpretability of the DCNN models is essential in the medical imaging field. The DCNN model results were visualized for interpretation using the Grad-CAM technique. The superimposed heatmaps generated via Grad-CAM yielded additional information useful in clinical practice use-cases. These Grad-CAM results reflected the microcalcification regions that most affected DCNN model predictions.

Although the applicability of DCNN models to classify suspicious microcalcification was demonstrated herein, the limitations of this study should be considered. First, a relatively small population was studied. Although fivefold data augmentation of the training data was performed and the pre-trained networks were adapted to address this limitation, the potential risk in the training may remain. Second, the data analyzed were localized to a single hospital center. Additional investigations involving larger and multi-center populations are necessary for further analysis and improvement of the DCNN model performances, particularly in terms of their PPV. Third, the sensitivities of the models were low compared to the other performance-measure values. This problem may be resolved if more data were used for training. Finally, our study population only sampled the patients recommended for biopsy owing to suspicious microcalcifications. However, a biopsy may not have been recommended for all patients when one was warranted; hence, they may have been excluded from the population despite their eligibility. This selection bias can limit the generalizability of the results obtained.

## Materials and methods

### Study subjects

The institutional review board (IRB) of Gangnam Severance Hospital (Approval Number: 3-2018-0176) approved this retrospective, single-center study and the written informed consent of patients was waived. All research was performed in accordance with relevant guidelines and regulations.

The cohort comprised patients exhibiting suspicious microcalcifications in screening mammograms, who underwent stereotactic vacuum-assisted biopsy (SVAB) or surgical excisional biopsy for histopathologic confirmation between July 2007 and December 2019. Our study focused only on microcalcifications, and the microcalcification lesions associated with masses were excluded. Furthermore, patients with breast related symptoms, a history of breast cancer, previous breast operations, and implants, and without histopathologic results, were excluded.

For each patient, the mammography examination was considered the index examination, followed by histopathologic confirmation, during our study period. For the final analysis, 1579 mammograms from 821 patients were utilized. The dataset was split into three distinct sets. For training and validating the DCNN models, 1319 mammograms obtained from 676 patients between 2007 and 2017 were used. The test dataset consisted of 260 mammograms obtained from 145 patients between 2018 and 2019.

All mammographic images were obtained via a full-field digital mammography unit (Lorad Selenia, Hologic, Danbury, CT, USA). Standard craniocaudal and mediolateral oblique views were obtained for both breasts along with spot-magnification views over the microcalcification regions.

### DCNN architectures

Over the past few years, DCNN performance has drastically improved on increasing network depth. In this study, we use five state-of-the-art DCNN architectures, ResNet-101^16^, Xception^17^, Inception-v3^18^, Inception-ResNet-v2^19^, and DenseNet-201^20^, which were pre-trained on the ImageNet dataset, to implement transfer learning from natural images to microcalcification images. The features of the five DCNN models are described in the Supplementary materials.

The classifier layers in the DCNN models were replaced with a new classifier for microcalcification characterization. To this end, the weights of the existing convolution layers were initialized with the weights of the pre-trained DCNN models, whereas new classifier layers were initialized with random weights. Subsequently, the parameters of the convolution and classifier layers were fine-tuned through backpropagation using the microcalcification-image dataset.

### DCNN model fine-tuning

Model fine-tuning was performed on a Windows 10 personal computer (PC) with an NVIDIA GTX 2080Ti graphics processing unit (GPU). The five DCNN models were implemented in MATLAB R2020a using the Deep Learning Toolbox.

#### Data preparation

Of the 1579 mammographic images, 1121 (positive, 418; negative 703) were randomly chosen as training data and 198 (positive, 74; negative, 124) as validation data. The remaining images (positive, 97; negative, 163) were independently selected as test data. To generate the target classification outcomes in each dataset, a radiologist with more than 10 years of experience in mammography cropped each lesion by applying a square region of interest (ROI) of 512 × 512 pixels using routines written in MATLAB. Each cropped image was classified as either benign or malignant and accordingly saved in the corresponding folder. Data augmentation of the training dataset was used to avoid overfitting and increase the number of training examples, because deep learning shows better results with more data. The 1121 training images were randomly flipped horizontally and vertically, rotated between 1° and 359°, and translated along the x- and y-axis by − 30 to + 30 pixels. The validation and test data were not augmented.

#### Training

When fine-tuning the pre-trained network, we used the adaptive moment estimation (Adam) optimizer, which combines the advantages of the adaptive gradient algorithm (AdaGrad) and root mean square propagation (RMSProp) techniques. The Adam optimizer (${\beta }_{1}=0.9$ and ${\beta }_{2}=0.999$) was used with an initial learning rate of 1e−4 for fine-tuning the five DCNNs. To ensure that the DCNN models avoid overfitting and generalize well, we used L2 regularization (weight decay) to penalize large weights. The hyper-parameter for controlling the amount of the regularization was set to 0.0005 for all DCNN models. Furthermore, we adopted an early stopping strategy to monitor the validation loss. The “patience,” i.e., the number of epochs that the model waits for to observe loss reduction in the validation set before stopping the training, was set to 20. The mini-batch sizes were set to 32 for Xception and Inception-v3, 16 for ResNet-101 and Inception-ResNet-v2, and 8 for DenseNet-201. These sizes were determined based on the maximum capacity of the GPU. The best DCNN models were selected based on the best AUCs obtained on the validation set.

### Interpretation of DCNN models via Grad-CAM

To understand the basis of classification decisions made by the five DCNN models, we employed the Grad-CAM technique^14^. Grad-CAM utilizes the gradient of the convolutional-feature-based classification score determined by the network to identify the most critical areas of the image for classification. Areas with large gradients are the ones where the final score depends most on the data. The Grad-CAM visualized the image results via heatmaps.

### Ensemble DCNN model

Several strategies exist for combining outputs from different models^22^. For example, the feature vectors from different models can be concatenated and a single classifier can be trained using the resulting higher-dimensional input. Another approach involves computing the average of the model outputs; this strategy was used herein to train the five DCNN models separately and average their predictions for testing.

### Data and statistical analysis

Histopathologic results obtained via SVAB or excisional biopsy served as reference standards for the imaging findings. Ductal carcinoma in situ (DCIS) and invasive carcinoma were classified as malignant. All other final histopathologic results, including high-risk lesions, were classified as benign. To evaluate the discriminative power of the DCNN models, the following quantitative measures were calculated: overall classification accuracy (ACC), sensitivity (SENS), specificity (SPEC), PPV, NPV, and AUC. The best cut-off point for each DCNN model to differentiate between malignant and benign microcalcification was set based on the maximal Youden index (sensitivity + specificity − 1)^23^.

The GEE method was used for comparing the diagnostic performances of the individual DCNN models, as well as the ensemble model, in terms of accuracy, sensitivity, specificity, PPV, and NPV^24^. We applied the non-parametric DeLong test of differences to the six AUCs^25^. The statistical analysis was performed using SAS software (version 9.4; SAS Institute, Cary, NC). A *p* < 0.05 value was considered to indicate a statistically significant difference.

## Supplementary Information

Supplementary Information.
