# Supplementary material for: A convolutional deep learning model for improving mammographic breast-microcalcification diagnosis
Source: Sci Rep. 2021 Dec 14;11:23925. doi: 10.1038/s41598-021-03516-0 (PMC8671560; doi:10.1038/s41598-021-03516-0)
Supplement: Supplementary file 1 — Supplementary Information. [file 41598_2021_3516_MOESM1_ESM.docx]

Supplementary

The features of 5 pre-trained DCNN models as follows:

***ResNet-101.*** ResNet is a residual network which can capture the discriminative features in a powerful way as well as avoid a problem on vanishing gradients of very deep CNNs. CNN models prior to ResNet learned features at different abstraction levels at the end of each convolution layer. Rather than learning features, ResNet learns residuals, which is the subtraction of learned features from input for each convolution layer and assists in avoiding the degradation problem that occurs for very deep CNNs. This is done by using a concept called identity shortcut connections. In this study, we used 101 layers.

***Xception.*** Xception uses depth wise separable convolution to involve the spatial dimension and channel dimension of the image separately in the training process replacing inception blocks. Xception has almost the same number of parameters as InceptionV3 with slightly better performance on ImageNet.

***InceptionV3.*** Inception network uses many different network filter sizes and pooling layers in parallel in inception block. The outputs of network are concatenated and transmitted to the next inception block. Furthermore, Inception utilizes a factorization method to improve the computational complexity of convolution layers as well as to make a network deeper and wider. The Inception network used in this study was InceptionV3.

***InceptionResnetV2.*** InceptionResnet is a hybrid model which combines inception and residual blocks. Inception block effectively learns features at different resolutions within the same convolution layer, while residual block enables the network to have deeper CNN to learn features that are more complex without losing performance. In this study, we implemented InceptionResnetV2 which combines InceptionV4 and ResNet architectures.

***DenseNet.*** ResNet significantly changed the view of how to parametrize the functions in deep networks. DenseNet is to some extent the logical extension of the ResNet. Dense block is the features of DenseNet where receives the output of all preceding layers as input and passes its own output to all subsequent layers. Therefore, each layer obtains the collective knowledge of all preceding layers. The resulting CNN model becomes thinner and more compact due to the decreasing number of feature maps all the input layers obtain the output from the previous layers.

Table S1. Diagnostic performance among DCNN models (learning rate = 1e-5)

| DCNN models | Cut-off  point | AUC  (95% CI) | Sensitivity  (95% CI) | Specificity  (95% CI) | Accuracy  (95% CI) | PPV  (95% CI) | NPV  (95% CI) |
| --- | --- | --- | --- | --- | --- | --- | --- |
| ResNet-101 | >0.122 | 0.798  (0.738-0.858) | 69.07  (59.87-78.27) | 83.44  (77.73-89.15) | 78.08  (73.05-83.11) | 71.28  (62.13-80.43) | 81.93  (76.08-87.78) |
| Xception | >0.389 | 0.798  (0.740-0.856) | 61.86  (52.19-71.53) | 85.28  (79.84-90.72) | 76.54  (71.39-81.69) | 71.43  (61.77-81.09) | 78.98  (72.96-85.00) |
| Inception-v3 | >0.391 | 0.719  (0.654-0.784) | 62.89  (53.28-72.50) | 72.39  (65.53-79.25) | 68.85  (63.22-74.48) | 57.55  (48.14-66.96) | 76.62  (69.94-83.30) |
| Inception Resnet-v2 | >0.242 | 0.853  (0.803-0.903) | 67.01  (57.65-76.37) | 91.41  (87.11-95.71) | 82.31  (77.67-86.95) | 82.28  (73.86-90.70) | 82.32  (76.76-87.88) |
| DenseNet-201 | >0.078 | 0.786  (0.729-0.843) | 74.23  (65.53-82.93) | 72.39  (65.53-79.25) | 73.08  (67.69-78.47) | 61.54  (52.72-70.36) | 82.52  (76.30-88.74) |
| Ensemble | >0.373 | 0.820  (0.763-0.876) | 58.76  (48.96-68.56) | 95.71  (92.60-98.82) | 81.92  (77.24-86.60) | 89.06  (81.41-96.71) | 79.59  (73.95-85.23) |
| p-value |  | <.0001 | 0.0050 | <.0001 | <.0001 | <.0001 | 0.0604 |

TableS2. pairwise comparison between two DCNN models (p-value): learning rate = 1e-4

| **AUC** | ResNet-101 | Xception | Inception-v3 | Inception Resnet-v2 | DenseNet-201 |
| --- | --- | --- | --- | --- | --- |
| ResNet-101 |  |  |  |  |  |
| Xception | 0.3274 |  |  |  |  |
| Inception-v3 | **0.0492** | 0.2042 |  |  |  |
| Inception Resnet-v2 | 0.9659 | 0.2955 | **0.0394** |  |  |
| DenseNet-201 | 0.8286 | 0.5742 | 0.1401 | 0.7712 |  |
| Ensemble | 0.2596 | **0.0065** | **<.0001** | 0.1365 | 0.2133 |

| **Accuracy** | ResNet-101 | Xception | Inception-v3 | Inception Resnet-v2 | DenseNet-201 |
| --- | --- | --- | --- | --- | --- |
| ResNet-101 |  |  |  |  |  |
| Xception | 0.5046 |  |  |  |  |
| Inception-v3 | 0.1387 | 0.3419 |  |  |  |
| Inception Resnet-v2 | 0.3164 | 0.6546 | 0.6681 |  |  |
| DenseNet-201 | **0.0172** | 0.0651 | 0.2320 | 0.1007 |  |
| Ensemble | 0.8618 | 0.4909 | 0.0701 | 0.2722 | **0.0115** |

| **Sensitivity** | ResNet-101 | Xception | Inception-v3 | Inception Resnet-v2 | DenseNet-201 |
| --- | --- | --- | --- | --- | --- |
| ResNet-101 |  |  |  |  |  |
| Xception | 0.7962 |  |  |  |  |
| Inception-v3 | **0.0031** | **0.0004** |  |  |  |
| Inception Resnet-v2 | **0.0152** | **0.0099** | 0.5630 |  |  |
| DenseNet-201 | **0.0009** | **0.0009** | 0.2481 | 0.0848 |  |
| Ensemble | 0.0848 | **0.0114** | 0.0542 | 0.2537 | **0.0217** |

| **Specificity** | ResNet-101 | Xception | Inception-v3 | Inception Resnet-v2 | DenseNet-201 |
| --- | --- | --- | --- | --- | --- |
| ResNet-101 |  |  |  |  |  |
| Xception | 0.2735 |  |  |  |  |
| Inception-v3 | **<.0001** | **0.0011** |  |  |  |
| Inception Resnet-v2 | **0.0017** | **0.0315** | 0.4101 |  |  |
| DenseNet-201 | **<.0001** | **<.0001** | **0.0210** | **0.0013** |  |
| Ensemble | **0.0429** | 0.4044 | **0.0006** | 0.0578 | **<.0001** |

| **PPV** | ResNet-101 | Xception | Inception-v3 | Inception Resnet-v2 | DenseNet-201 |
| --- | --- | --- | --- | --- | --- |
| ResNet-101 |  |  |  |  |  |
| Xception | 0.3092 |  |  |  |  |
| Inception-v3 | **0.0005** | **0.0170** |  |  |  |
| Inception Resnet-v2 | **0.0120** | 0.1247 | 0.5136 |  |  |
| DenseNet-201 | **<.0001** | **0.0002** | 0.0864 | **0.0153** |  |
| Ensemble | 0.1310 | 0.709 | **0.0043** | 0.1023 | **<.0001** |

| **NPV** | ResNet-101 | Xception | Inception-v3 | Inception Resnet-v2 | DenseNet-201 |
| --- | --- | --- | --- | --- | --- |
| ResNet-101 |  |  |  |  |  |
| Xception | 0.9715 |  |  |  |  |
| Inception-v3 | 0.0736 | **0.0232** |  |  |  |
| Inception Resnet-v2 | 0.1301 | 0.0775 | 0.7588 |  |  |
| DenseNet-201 | 0.0540 | **0.0441** | 0.5383 | 0.3587 |  |
| Ensemble | 0.2015 | **0.0331** | 0.3676 | 0.6349 | 0.2445 |

TableS3. pairwise comparison between two DCNN models (p-value): learning rate = 1e-5

| **AUC** | ResNet-101 | Xception | Inception-v3 | Inception Resnet-v2 | DenseNet-201 |
| --- | --- | --- | --- | --- | --- |
| ResNet-101 |  |  |  |  |  |
| Xception | 0.9926 |  |  |  |  |
| Inception-v3 | **0.0060** | **0.0016** |  |  |  |
| Inception Resnet-v2 | **0.0295** | **0.0036** | **<.0001** |  |  |
| DenseNet-201 | 0.6309 | 0.6161 | **0.0175** | **0.0069** |  |
| Ensemble | 0.2421 | 0.1101 | **<.0001** | **0.0335** | 0.0869 |

| **Accuracy** | ResNet-101 | Xception | Inception-v3 | Inception Resnet-v2 | DenseNet-201 |
| --- | --- | --- | --- | --- | --- |
| ResNet-101 |  |  |  |  |  |
| Xception | 0.5551 |  |  |  |  |
| Inception-v3 | **0.0036** | **0.0127** |  |  |  |
| Inception Resnet-v2 | 0.1363 | **0.0208** | **<.0001** |  |  |
| DenseNet-201 | 0.0833 | 0.2704 | 0.1903 | **0.0036** |  |
| Ensemble | 0.1030 | **0.0073** | **<.0001** | 0.8474 | **0.0027** |

| **Sensitivity** | ResNet-101 | Xception | Inception-v3 | Inception Resnet-v2 | DenseNet-201 |
| --- | --- | --- | --- | --- | --- |
| ResNet-101 |  |  |  |  |  |
| Xception | 0.0848 |  |  |  |  |
| Inception-v3 | 0.1970 | 0.8348 |  |  |  |
| Inception Resnet-v2 | 0.6695 | 0.2481 | 0.3436 |  |  |
| DenseNet-201 | 0.2481 | **0.0154** | **0.0368** | 0.1739 |  |
| Ensemble | **0.0055** | 0.3637 | 0.3148 | **0.0285** | **0.0005** |

| **Specificity** | ResNet-101 | Xception | Inception-v3 | Inception Resnet-v2 | DenseNet-201 |
| --- | --- | --- | --- | --- | --- |
| ResNet-101 |  |  |  |  |  |
| Xception | 0.5771 |  |  |  |  |
| Inception-v3 | **0.0080** | **0.0009** |  |  |  |
| Inception Resnet-v2 | **0.0215** | **0.0387** | **<.0001** |  |  |
| DenseNet-201 | **0.0027** | **0.0007** | >.9999 | **<.0001** |  |
| Ensemble | **<.0001** | **<.0001** | **<.0001** | **0.0495** | **<.0001** |

| **PPV** | ResNet-101 | Xception | Inception-v3 | Inception Resnet-v2 | DenseNet-201 |
| --- | --- | --- | --- | --- | --- |
| ResNet-101 |  |  |  |  |  |
| Xception | 0.9732 |  |  |  |  |
| Inception-v3 | **0.0034** | **0.0031** |  |  |  |
| Inception Resnet-v2 | **0.0322** | **0.0215** | **<.0001** |  |  |
| DenseNet-201 | **0.0162** | **0.0276** | 0.3165 | **<.0001** |  |
| Ensemble | **<.0001** | **<.0001** | **<.0001** | 0.1093 | **<.0001** |

| **NPV** | ResNet-101 | Xception | Inception-v3 | Inception Resnet-v2 | DenseNet-201 |
| --- | --- | --- | --- | --- | --- |
| ResNet-101 |  |  |  |  |  |
| Xception | 0.1441 |  |  |  |  |
| Inception-v3 | **0.0352** | 0.3374 |  |  |  |
| Inception Resnet-v2 | 0.8646 | 0.1024 | **0.0111** |  |  |
| DenseNet-201 | 0.8078 | 0.1823 | **0.0486** | 0.9429 |  |
| Ensemble | 0.1735 | 0.6811 | 0.1425 | 0.0935 | 0.1946 |
